# Involvement of DDAH/ADMA/NOS/cGMP and COX-2/PTGIS/cAMP Pathways in Human Tissue Kallikrein 1 Protecting Erectile Function in Aged Rats

**DOI:** 10.1371/journal.pone.0170427

**Published:** 2017-01-19

**Authors:** Kai Cui, Yang Luan, Zhe Tang, Ke Rao, Tao Wang, Zhong Chen, Shaogang Wang, Jihong Liu, Daowen Wang

**Affiliations:** 1 Department of Urology, Tongji Hospital, Tongji Medical College, Huazhong University of Science and technology, Wuhan, Hubei, China; 2 Institute of Urology, Tongji Hospital, Tongji Medical College, Huazhong University of Science and Technology, Wuhan, Hubei, China; 3 Division of Cardiology, Department of Internal Medicine, Tongji Hospital, Tongji Medical College, Huazhong University of Science and Technology, Wuhan, Hubei, China; The Chinese University of Hong Kong, HONG KONG

## Abstract

Our previous studies had reported that Human Tissue Kallikrein 1 (hKLK1) preserved erectile function in aged transgenic rats, while the detailed mechanism of hKLK1 protecting erectile function in aged rats through activation of cGMP and cAMP was not mentioned. To explore the latent mechanism, male wild-type Sprague-Dawley rats (WTR) and transgenic rats harboring the hKLK1 gene (TGR) were fed to 4 and 18 months old and divided into four groups: young WTR (yWTR) as the control, aged WTR (aWTR), aged TGR (aTGR) and aged TGRs with HOE140 (aTGRH). Erectile function of all rats was evaluated by cavernous nerve electrostimulation method and measured by the ratio of intracavernous pressure/ mean arterial pressure (ICP/MAP) in rats. Expression levels of cAMP and cGMP were assessed, and related signaling pathways were detected by western blot, immunohistochemistry and RT-PCR. Our experiment results showed erectile function of the aWTR group and aTGRH group was lower compared with those of other two groups. Also, expression levels of cAMP and cGMP were significantly lower than those of other two groups. Moreover, expressions of related signaling pathways including DDAH/ADMA/NOS/cGMP and COX-2/PTGIS/cAMP were also downregulated in the corpus cavernosum of rats in aWTR group. Our finding revealed hKLK1 played a protective role in age-related ED. The DDAH/ADMA/NOS/cGMP and COX-2/PTGIS/cAMP pathways that were linked to the mechanism hKLK1 could increase the levels of cGMP and cAMP, which might provide novel therapy targets for age-related ED.

## Introduction

Erectile dysfunction (ED), defined as an inability to attain or maintain sufficient penile erection for satisfactory sexual intercourse, is one of the most frequent conditions in andrology [1]. ED has various etiologies, including many risk factors of vascular diseases, neurologic abnormalities, and hormonal disturbances [2,3]. Aging is one of the most common risk factors for male sexual dysfunction, and age-related ED may seriously affect the quality of life in men aged above 40 years. Previous epidemiological studies have also shown that ED was a complex disorder, with aging as an independent predictor [4]. Recent epidemiological studies demonstrated that the prevalence of ED ranged from 2% to 9% in men aged 40–49 years, and increased to 20–40% in men aged 60–69 years, and affected almost all the men older than 70 years [5–7]. Age-related ED is difficult to treat effectively with conventional drugs [8], wherefore a better understanding of age-related ED is urgently needed to facilitate the development of new therapy strategies.

Nitric oxide (NO) is generated by three different isoforms of enzyme nitric oxide synthase (NOS), endothelial NOS (eNOS), neuronal NOS (nNOS) and inducible NOS (iNOS) [9], among which eNOS and nNOS are strongly associated with ED [10]. NO induces the activation of soluble guanylyl cyclase and the accumulation of cyclic guanosine monophosphate (cGMP), resulting in smooth muscle relaxation and penile erection [11]. Asymmetric dimethylarginine (ADMA) is a powerful inhibitor of all three types of NOS, and can be degraded into citrulline and dimethylarginine by dimethylarginine dimethylaminohydrolase (DDAH), which predominates in tissue expressing NOS [12]. Wang *et al*. reported on the role of the DDAH/ADMA/NOS/cGMP pathway in erectile function of rats, while whether it was involved in the process of age-related ED in rats remained unclear [13].

In addition to the NO/cGMP pathway, cyclic adenosine monophosphate (cAMP) concentration is also closely associated with ED [14]. Arachidonic acid (AA) can be converted to prostaglandin H2 (PGH2) by cyclooxygenase (COX) enzymes, including COX-1 and/or COX-2. Under the catalysis of prostacyclin synthase (PTGIS), PGH2 will change into prostacyclin (PGE2), followed by induction of cAMP production, cavernouse smooth muscle relaxation and penile erection [15]. Lin *et al*. reported that COX-2-10aa-PGIS gene therapy improved erectile function in cavernous nerve injured rat model [14]. Similarly, we previously reported testosterone ameliorate ED after castration, by increasing the activity of the COX-2/PTGIS/cAMP pathway [16]. However, whether the COX-2/PTGIS/cAMP pathway plays a similar role in aged rat remains to be explored.

Tissue kallikrein 1 (KLK1) is a glycoprotein of the serine proteinase superfamily that was initially discovered as a hypotensive agent in human urine. It processes low-molecular-weight kininogen (LMWK) to produce vasoactive kinins, which can exert biological functions such as reducing cardiac and renal injuries, restenosis and ischemic stroke, and promoting angiogenesis and skin wound healing via kinin receptor signaling [17]. Our previous work suggested *hKLK1* may preserve erectile function in aged rats via activation of eNOS/cGMP signaling [18]. However, whether DDAH/ADMA/eNOS and COX-2/PTGIS/cAMP pathways are involved in the mechanisms of hKLK1’s effect in age-related ED remain unclear.

## Materials and Methods

### Acquisition of the Transgenic Rat (TGR)

TGR, which was generated by microinjecting a 5.6 kb DNA fragment into oocytes of Sprague-Dawley (SD) rats under the control of the heavy-metalresponsive mouse metallothionein (mMT1) promoter as previous constructed [19,20]. Presence of the transgene in genomic DNA isolated from the rat tail was verified by Southern blotting, as described previously [20,21]. We need to thank the Max-Delbrück-Center for Molecular Medicine for the precious gift of the homozygous transgenic rats, which were used for the following experiments.

### Experimental Animals

All procedures were approved by the Institutional Animal Care and Use Committee of Tongji Hospital, Tongji Medical College, Huazhong University of Science and Technology (Hubei, China). 40 male SD rats were used, including 20 wild-type SD rats (WTRs) (Laboratory Animal Center of Tongji Medical College, Huazhong University of Science and Technology), and 20 TGRs. All the rats were bred by professional breeders under the same conditions until they were 4 months old (weighing 250–300 g) or 18 months old (weighing 450-500g).

The 40 rats were divided into four groups: young WTR group (yWTR, control group, 4-month-old, n = 10); aged WTR group (aWTR, 18-month-old, n = 10); aged TGR group (aTGR, 18-month-old, n = 10) and aged TGR group with HOE140 (100thol/kg.d; intraperitoneal injection for 2 weeks; aTGRH, 18-month-old, n = 10).

### Verification of TGR

In order to detect the expression of *hKLK1* gene in the penile tissues of rats, we used conventional polymerase chain reaction (PCR) and agarose gel electrophoresis, real-time reverse transcriptase-PCR (RT-PCR) and western blot to determine the hKLK1 in frozen corpus cavernosum samples at the level of DNA, mRNA and protein levels, respectively. The primer sequences are listed in Table 1.

**Table 1:** Primers used in conventional PCR and Real-time RT-PCR.

Genes	Primer sequences	Usage (PCR)
*hKLK1*	F: 5’-GTCCAGAAGGTGACAGACTTCAT-3’	Conventional
	R: 5’-GTCCTCGATCCACTTCACATAAG-3’	
	F: 5’-CTCACAGCTGCTCATTGCATC-3’	Real-time
	R: 5’-GCTCTCACTGACATGAACAAACTGG-3’	
*rKLK1*	F: 5’-CCCACACACAGATGGTGACAGA-3’	Real-time
	R: 5’-CCTTGAAGCACACCATCACAGAG-3’	
*COX-2*	F: 5’-TGAACACGGACTTGCTCACTTT-3’	Real-time
	R: 5’-AGGCCTTTGCCACTGCTTGTA-3’	
*PTGIS*	F: 5’-GCTATGCCATCAACAGCATCAAAC-3’	Real-time
	R: 5’-AGTGTAGTGTCTGCTCCACAGGTCA-3’	
*nNOS*	F: 5’- CCTATGCCAAGACCCTGTGTGA-3’	Real-time
	R: 5’- CATTGCCAAAGGTGCTGGTG-3’	
*eNOS*	F: 5’- GATCCTAACTTGCCTTGCATCCT-3’	Real-time
	R: 5’- TGTAATCGGTCTTGCCAGAATCC-3’	
*β-actin*	F: 5’- AAGAGCTATGAGCTGCCTGA-3’	Conventional & Real-time
	R: 5’- TACGGATGTCAACGTCACAC-3’	

### Measurement of Erectile Function *in vivo*

As described in our previous studies [18], all rats were anesthetized by intraperitoneal injection of pentobarbital (40 mg/kg). The carotid artery on the left side was exposed, and a PE-50 tube filled with heparinized saline (100 IU/ml) was cannulated into the artery and connected to a pressure transducer for continuous monitor of arterial pressure. The cavernous nerve on the left side was then separated, and a 25-G needle was inserted into the left penile crura to monitor intracavernous pressure (ICP). The cavernous nerve was electrostimulated at 5 volt, 15 HZ, with a pulse width of 1.2 milliseconds for 1 min and a 3-min interval before subsequent stimulation [22]. The ratios of both the maximal ICP and the area under the ICP curve (AUC) to mean arterial pressure (MAP) were calculated to evaluate erectile function *in vivo*.

### Real-Time RT-PCR

Total RNA was obtained from the corpus cavernosum using a Multisource Total RNA Miniprep Kit (AXYGEN, Union City, CA, USA) according to the manufacturer’s instructions. Reverse transcription and real-time PCR were conducted using PrimeScript RT Master Mix and SYBR Green PCR Master Mix (TaKaRa, Dalian, Liaoning, China), as described in our previous studies [23]. The mRNA expression levels of the examined genes relative to β-actin were calculated using the 2^-ΔCt^ method. The primer sequences used for *hKLK1*, rat tissue *KLK1 (rKLK1)*, eNOS, nNOS, COX-2, PTGIS and β-actin were shown in Table 1.

### Western Blot Analysis

Frozen penile tissues were isolated and prepared in the RIPA buffer containing a protease inhibitor cocktail and sodium fluoride, followed by centrifugation at 12,000 × g for 10 min at 4°C,as described in our previous studies [24]. Equal amounts (40μg/lane) of proteins were separated by sodium dodecyl sulfate-polyacrylamide gel electrophoresis and transferred to polyvinylidene fluoride membranes (Immobilon-P Transferred Membrane; Millipore Corporation, Billerica, MA, USA). After blocking in 5% bovine serum albumin for 1 h at room temperature, the membranes were incubated with antibodies against: hKLK1 (1:5000; Sigma Aldrich, St. Louis, MO, USA), rKLK1 (1:1000; Sigma Aldrich), COX-2 (1:500; Abcam, Cambridge, MA, USA), PTGIS (1:1000; Abcam), DDAH1 (1:1000; Abcam), DDAH2 (1:1000; Proteintech, Wuhan, Hubei, China), eNOS (1:1000; Abcam), P-eNOS (T495; 1:1000; Abcam), P-eNOS (S1177; 1:500; Abcam), nNOS (1:1000; Abcam) and β-actin (1:1000; Proteintech) overnight at 4°C.

After washing three times in TBST for 30 min, the membranes were incubated with horseradish peroxidase-conjugated secondary antibodies (1:5000; Proteintech) for 1 h followed by a further 30 min washing, Finally, the bands were analyzed using an enhanced chemiluminescence detection system (Pierce; Thermo Fisher Scientific, Rockford, IL, USA).

### Assessment of cGMP, cAMP and ADMA

cGMP and cAMP concentrations in the penile tissues were measured using an enzyme-linked immunosorbent assay (ELISA) kit (Nanjing Jiancheng Bioengineering Institute, Nanjing, Jiangsu, China) according to the manufacturer’s instructions, as described in our previous studies [16]. The concentration of ADMA in penile tissue was assessed using a commercial ELISA kit (Bio-Swamp Immunoassay R&D Center, Shanghai, China) according to the manufacturer’s instructions. The assays were performed in triplicate, and the total protein concentrations were detected to normalize the data.

### Histological Examinations

The locations and expressions levels of DDAH1, DDAH2, eNOS and nNOS were investigated by immunohistochemically staining of penile tissue sections (5 μm thickness) of corpus cavernosum. Sections were incubated with primary antibodies against: DDAH1 (1:100; Abcam), DDAH2 (1:50; Proteintech), eNOS (1:100; Abcam) and nNOS (1:100; Abcam) at 37°C for 1 h. Then a biotinylated secondary antibody was applied according to the standard protocol [23]. Semiquantitative analysis was performed to evaluate the staining intensity using Image-Pro plus 6.0 software (Media Cybernetics, Silver Spring, MD, USA).

### Statistical Analysis

Results were analyzed using GraphPad Prism version 5.0 (GraphPad Software, San Diego, CA, USA) and expressed as mean ± standard error of the mean. Statistical analyses were performed using one-way analysis of variance followed by the Tukey-Kramer test for *post hoc* comparisons. Differences among groups were considered significant at *P* < 0.05.

## Results

### hKLK1 Preserved Erectile Function of Aged Rats

**Fig 1:**
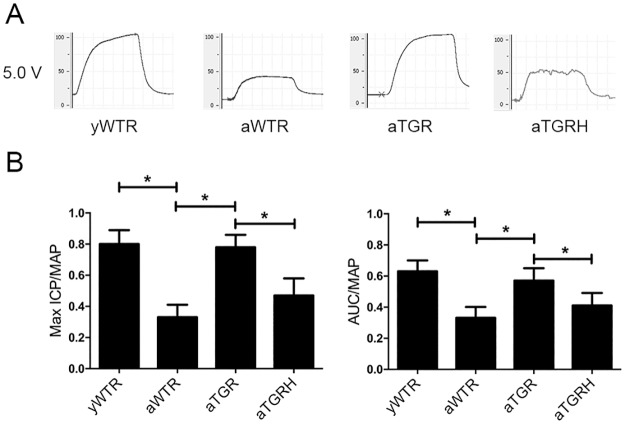
Erectile Function of all rats was measured through cavernous nerve electrostimulation. (A) Representative curves of ICP through the electrostimulation of 5V setting for 1 min. (B) Erectile function was evaluated by measuring the maximal ICP/MAP, and the AUC/MAP. The data are expressed as mean ± s.d. *P<0.05 when comparing the two groups under each end of the capped line. ICP: intracavernous pressure; MAP: mean arterial pressure; s.d.: standard deviation.

Erectile function was evaluated by measuring the maximal ICP/MAP, and the AUC/MAP at the stimulating voltages of 5V. Both the parameters were significantly lower in the aWTR group than those in the yWTR (*P*<0.05). However, hKLK1 could restore the erectile function of aTGR to normal level of the yWTR group, while the protective role was abolished in the aTGRH group (*P* >0.05) (Fig 1).

### Verification of TGR

**Fig 2:**
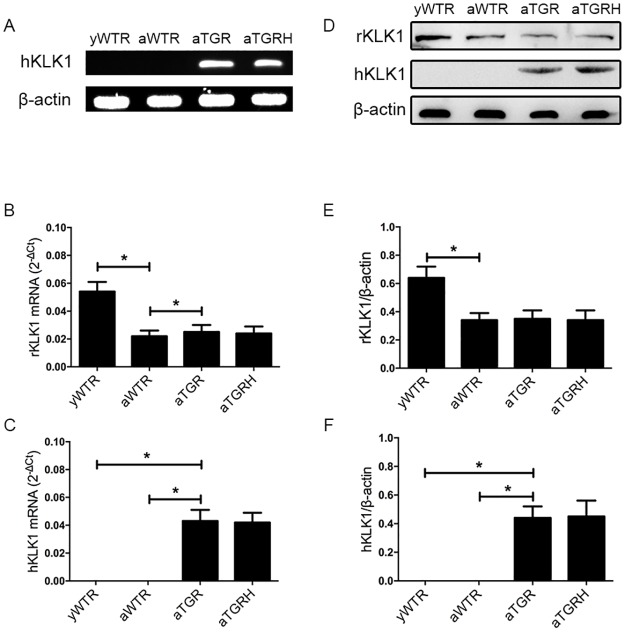
Verification of presence and expression of hKLK1 and rKLK1 genes in the corpus cavernosum. (A): Representative hKLK1 genomics DNA bands in rats’ corpus cavernosum by agarose gel electrophoresis followed by conventional PCR. (B) and (C): Relative mRNA expressions of hKLK1 and rKLK1 with β-actin as the loading control in corpus cavernosum of rats in all groups by RT-PCR using the 2-ΔCt method. (D), (E) and (F): Expressions of hKLK1 and rKLK1 in the corpus cavernosum of rats in all groups by western blot analysis. The data are expressed as mean ± s.d. *P<0.05 when comparing the two groups under each end of the capped line.

We detected the expression of the *hKLK1* gene in the corpus cavernosum of rats at the DNA, mRNA and protein levels, and we found that all rats in the aTGR and aTGRH groups carried the *hKLK1* gene. In addition, the *hKLK1* gene was confirmed to be absent in the yWTR and aWTR groups. Meanwhile, mRNA and protein expression levels of rKLK1 were lower in the aWTR group compared with the yWTR group (both *P* <0.05) (Fig 2).

### Levels of DDAH1, ADMA and nNOS in Rat Penis

**Fig 3:**
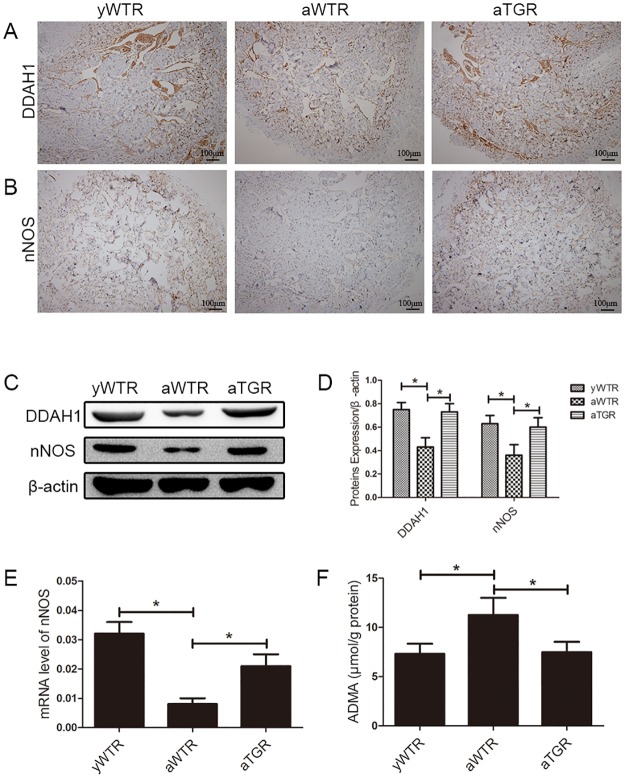
Expression levels of DDAH1, nNOS and ADMA. (A) and (B): Immunohistochemistry results of expressions of DDAH1 and nNOS (magnification × 100). (C): Representative western blot results of DDAH1 and nNOS in corpus cavernosum of three groups. (D): Expressions of DDAH1 and nNOS with β-actin as the loading control in the corpus cavernosum of three groups were presented through bar graphs. (E): nNOS expression level with β-actin as the loading control in penile tissues of all groups by real-time RT-PCR using the 2-ΔCt method. (F): ADMA concentrations of all groups assessed by ELISA method. The data are expressed as mean ± s.d. *P<0.05 when comparing the two groups under each end of the capped line.

According to the result of western blotting, protein levels of DDAH1 and nNOS in the aWTR group were significantly lower compared with the yWTR and aTGR group. In addition, there is almost no difference between the yWTR and aTGR groups. This is consistent with the result of immunohistochemistry. Moreover, ELISA was performed to assess the ADMA concentration. The ADMA concentration was higher in the aWTR group than that in both the yWTR and aTGR groups. Similarly, the aTGR group showed the same level of ADMA as the yWTR group (all *P* <0.05) (Fig 3).

### Expressions of the DDAH2, eNOS and cGMP in Rat Penis

**Fig 4:**
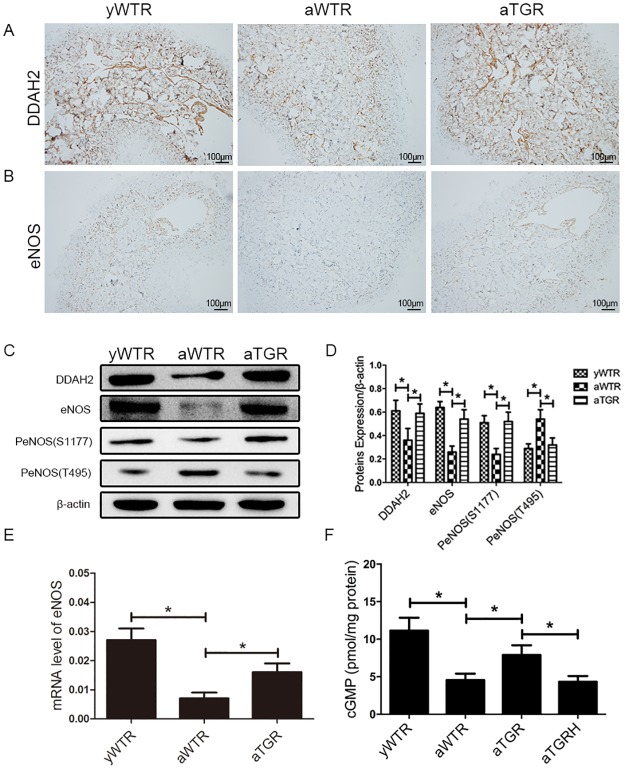
Expression levels of DDAH2, eNOS and cGMP. (A) and (B): Immunohistochemistry results of expressions of DDAH2 and eNOS (magnification × 100). (C) Representative western blot results of DDAH2, eNOS, P-eNOS (T495) and P-eNOS (S1177) in corpus cavernosum of all three groups. (D): Expressions of DDAH2, eNOS, P-eNOS (T495) and P-eNOS (S1177) with β-actin as the loading control in the corpus cavernosum of three groups were presented through bar graphs. (E): eNOS expression level in corpus cavernosum of rats in all groups by real—time RT-PCR using the 2-ΔCt method. (F): cGMP concentrations of all groups assessed by ELISA method. The data are expressed as mean ± s.d. *P<0.05 when comparing the two groups under each end of the capped line.

In our present study, western blotting analysis demonstrated that protein levels of DDAH2 and eNOS were also lower in the aWTR group compared with the yWTR and aTGR groups (all *P*<0.05). However, the aTGR group showed no significantly difference when compared to the yWTR group (both *P*>0.05). cGMP concentration in the cavernous tissue of the aWTR and aTGRH groups were also lower than that in the yWTR and aTGR groups, based on the ELISA results (all *P* <0.05). Moreover, no significantly difference was found between the yWTR group and the aTGR group at the concentration of cGMP (*P*>0.05) (Fig 4).

### hKLK1 Activates the COX-2/PTGIS/cAMP Pathway in Corpus Cavernosum of Aged Rats

**Fig 5:**
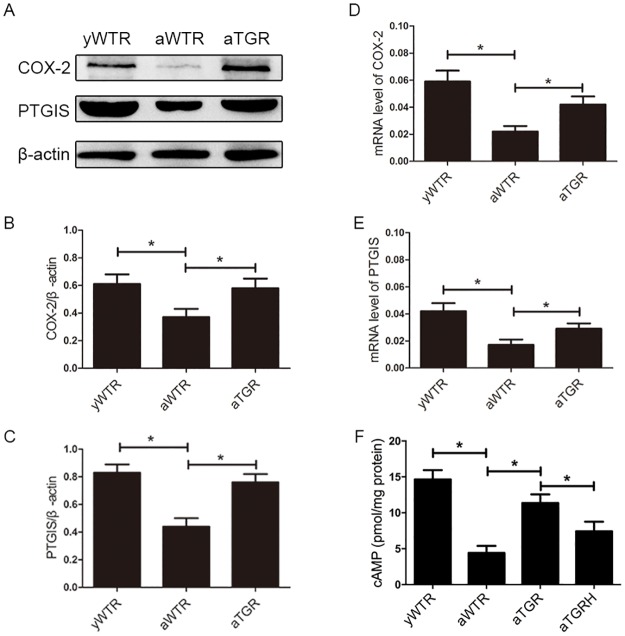
Expression levels of COX-2, PTGIS and cAMP. (A): Representative western blot results of COX-2 and PTGIS in corpus cavernosum of three groups. (B) and (C): Expressions of COX-2 and PTGIS with β-actin as the loading control in the corpus cavernosum of three groups were presented through bar graphs. (D) and (E): Relative mRNA level in corpus cavernosum of rats in all groups by RT-PCR using the 2-ΔCt method. (F): cAMP concentrations of all groups assessed by ELISA method. The data are expressed as mean ± s.d. *P<0.05 when comparing the two groups under each end of the capped line.

In our present study, results of Western blotting analysis showed expression levels of COX-2 and PTGIS were lower in the aWTR group compared with the yWTR and aTGR groups (all *P*<0.05). However, there is almost no difference between the yWTR and aTGR groups, which showed that the hKLK1 could restore the activation of COX-2/PTGIS pathway. cAMP concentration in the aWTR and aTGRH groups were also lower than in the yWTR and aTGR groups, based on ELISA results (all *P* <0.05). Similarly, no significant difference was found between the yWTR and aTGR groups (*P*>0.05) (Fig 5).

## Discussion

We previously demonstrated that the *hKLK1* gene plays a preventive role in age-related ED by promoting the expression of eNOS and inhibiting corporal fibrosis [18]. However, the detail mechanism of NOS regulation by *hKLK1* is still unknown. Furthermore, although cAMP is known to be benefit in age-related ED, few studies involved its regulatory way. The current study initially demonstrated that enhancing KKS by over-expression of *hKLK1* could regulate the activity of DDAH/ADMA/NOS/cGMP and the COX-2/PTGIS/cAMP pathways, which increased the production of cGMP and cAMP, resulting in improved age-related ED.

Normal penile erection includes first to initiate, followed by relaxation and maintenance of smooth muscle tone, in keeping with blood flow into the corpus cavernosum [3]. Any factor affecting relaxation of the expansion of the sinusoidal system and corporal smooth muscle would thus result in inadequate blood flow into the cavernous sinusoids, leading to reduced rigidity and ED [3,25].

NO is synthesized mainly from L-arginine by NOS in endothelial cells and plays an important role in maintaining the integrity of vascular structure and function, and is often described as an ‘endogenous anti-atherosclerotic molecule’ [26]. The production and release of NO by non-adrenergic, non-cholinergic nerves and the endothelial cells lining the corporal sinusoids have been shown to be the major mediator to induce cavernous smooth muscle relaxation via activating NO/cGMP signal transduction pathway [3,11]. Previous studies also revealed that reduced NOS activity was associated with age-related ED [2]. Together, there were many studies on the change of the NO/cGMP pathway in varieties of animal models, while few studies involved the upstream factors of NO/cGMP, which could play an important role in the expressions of NOS, NO and cGMP.

ADMA is a naturally occurring L-arginine analogue that is released into the cytoplasm following the post-translational methylation of arginine residues within proteins and the subsequent proteolysis of these arginine-methylated proteins. Moreover, ADMA has been reported to inhibit NOS activity and relaxation of blood vessels in response to acetylcholine, as an important endogenous modulator of vascular tone, under both resting and vasoactive stimuli conditions [27]. Similarly, Abhary *et al*. reported ADMA was a powerful inhibitor of all three types of NOS, through competition with L-arginine to bind to the active site of NOS [12]. Consequently, increase levels of circulating ADMA is closely linked to endothelial dysfunction in some cardiovascular abnormalities [28,29]. In addition, ADMA can be degraded into citrulline and dimethylamine by DDAH. DDAH includes two isoforms (DDAH1, DDAH2), of which DDAH1 predominates in penile tissue expressing nNOS, and DDAH2 predominates in penile tissue expressing eNOS [30]. Growing evidence suggests that decreased expression of DDAH might be a vital factor to induce formation of ADMA in some pathological conditions [31,32]. Wang *et al*. [13] reported the decreased expression of DDAH isoforms and accumulation of ADMA might synergistically decrease NO production and induce age-related ED. In our present study, we detected the activity of DDAH/ADMA/NOS/cGMP pathway to evaluate its role in hKLK1’s protective effect on age-related ED. The NOS/cGMP was inhibited in the aged wild-type group, while hKLK1 dramatically activated it by increasing the expression of DDAH isoforms, nNOS, eNOS, and cGMP, and decreasing ADMA level in the aTGR group. Moreover, the cGMP level was reduced in the aTGRH group when faced with the hKLK1’s protective role was inhibited. Taken together, our results showed that the DDAH/ADMA/NOS/cGMP pathway, the upstream factors of the well-recognized NO/cGMP pathway, was involved in the mechanism that *hKLK1* preserved erectile function in aged rats.

Except for cGMP, cAMP is also implicated in erectile physiology through the COX-2/PTGIS pathway [33,14]. COX enzymes include COX-1 and COX-2: COX-1 is constitutively expressed in cells, while COX-2 is expressed under certain anomalous conditions [26]. COX-2 and PTGIS are the two key enzymes in endothelial cells to produce PGE2, which can enter into corpus cavernosum smooth muscle cells to increase the cAMP level and induce the relaxation of smooth muscle. Similarly, the use of nonsteroidal anti-inflammatory drugs (NSAIDs) increases the risk of ED by inhibiting the expression of COX-2 and reducing the production of PGE2, thereby decreasing cAMP expression [34]. Lin *et al*. reported *COX-2-10aa-PGIS* gene therapy improved ED caused by cavernous nerve injury [14]. Previously, we also showed that the COX-2/PTGIS/cAMP pathway was involved in the pathogenesis of testosterone deficiency related ED, which suggests its potential role in age-related ED [16]. Therefore, cAMP was also an important factor in the physiological and pathological processes of erectile function as well as cGMP, while less articles mentioned the role of cAMP and its upstream factors COX-2 and PTGIS. In our present study, we verified that in the aWTR group the COX-2/PTGIS pathway was inhibited and the concentration of cAMP was reduced, while hKLK1 activated the signaling pathway and preserved their levels as same as those in the yWTR group. Moreover, the cAMP level was reduced again in the aTGRH group when the role of hKLK1 was inhibited. In conclusion, our results demonstrated that the COX-2/PTGIS/cAMP pathway was involved in the mechanism that hKLK1 protected erectile function in aged rats.

Tissue KLK1 is as a serine proteinase synthesized in many organs, and it can cleave LMWK into kinin peptides. Kinins exert a variety of biological effects, including antithrombotic and vasodilation effects, through binding to bradykinin 2 receptor (B2R). Becker *et al*. reported that BK had a relaxation effect on corpus cavernosum smooth muscle based on the release of NO, demonstrating that KKS might exert a vital role in penile erection [35]. It was also demonstrated that KLK1 expression changed in an age-dependent pattern in rat corpus cavernosum, with a downward trend during aging [36]. In order to systematically explore the protective roles of hKLK1 in multiple cardiovascular disease, Sliva *et al*. established a TGR model harboring *hKLK1* gene with overactive KKS, which could be used to study multiple issues of cardiovascular regulation and other physiological/pathological mechanisms that might involve kinins [21,37]. In our experiment results, we found that the expression of rKLK1 in the aWTR and aTGR groups were lower than the yWTR group, and the erectile function was almost the same level as the yWTR group, which suggested that the age-related ED was associated with the decrease of rKLK1, and the existence of the hKLK1 might play the same protective role as the rKLK1. In addition, the result of erectile function, cGMP and cAMP in the aTGRH group further supported our conclusion. With the TGR model, we found that *hKLK1* preserved erectile function in aged rats via activation of eNOS/cGMP, inhibition of RhoA/ROCK pathways, and anti-tissue fibrotic effects in the corpus cavernosum [18]. At the beginning of this current study, we confirmed the presence and expression of *hKLK1* in the corpus cavernosum at the DNA, mRNA, and protein levels, demonstrating the suitability of the TGR rat model. The changes of ICP/MAP and AUC/MAP in all four groups (yWTR, aWTR, aTGR, aTGRH) suggested the age-related decrease of penile response to cavernous nerve stimulation, and showed that *hKLK1* could preserve erectile function in aged TGRs. Further experiments elucidated the underlying mechanisms of the DDAH/ADMA/NOS/cGMP and COX-2/PTGIS/cAMP pathways.

This study revealed that the DDAH/ADMA/NOS and COX-2/PTGIS/cAMP pathways were involved in the mechanism that *hKLK1* could increase the expression of cGMP and cAMP. However, there were some limitations, including the lack of cellular based experiments. Blocking the pathways using specific inhibitors and RNA interference could provide further evidence to support our conclusions. Further studies are therefore needed to examine the effect of *hKLK1 in vitro* and to explore the molecular changes associated with its protective role in age-related ED.

## Conclusion

We concluded that hKLK1 plays a protective role in age-related ED. The DDAH/ADMA/NOS/cGMP and COX-2/PTGIS/cAMP pathways were linked to the mechanism hKLK1 could increase the levels of cGMP and cAMP, which might provide novel therapy targets for age-related ED.
